# Improved Ferroelectric Performance of Mg-Doped LiNbO_3_ Films by an Ideal Atomic Layer Deposited Al_2_O_3_ Tunnel Switch Layer

**DOI:** 10.1186/s11671-019-2970-6

**Published:** 2019-04-16

**Authors:** Yan Zhang, Qing Hua Ren, Xiao Jie Chai, Jun Jiang, Jian Guo Yang, An Quan Jiang

**Affiliations:** 1 State Key Laboratory of ASIC & System, School of Microelectronics, Fudan University, Shanghai, 200433 China; 2 State Key Laboratory of Functional Materials for Informatics, Shanghai Institute of Microsystem and Information Technology, Chinese Academy of Sciences, Shanghai, 200050 China

**Keywords:** Tunnel switch, Mg-doped LiNbO_3_, Atomic layer deposition, Ion slicing, Ferroelectric memory

## Abstract

Bilayer structures composed of 5% Mg-doped LiNbO_3_ single-crystal films and ultrathin Al_2_O_3_ layers with thickness ranging from 2 to 6 nm have been fabricated by using ion slicing technique combined with atomic layer deposition method. The transient domain switching current measurement results reveal that the *P-V* hysteresis loops are symmetry in type II mode with single voltage pulse per cycle, which may be attributed to the built-in electric field formed by asymmetric electrodes and compensation of an internal imprint field. Besides, the inlaid Al_2_O_3_, as an ideal tunnel switch layer, turns on during ferroelectric switching, but closes during the post-switching or non-switching under the applied pulse voltage. The Al_2_O_3_ layer blocks the adverse effects such as by-electrode charge injection and improves the fatigue endurance properties of Mg-doped LiNbO_3_ ferroelectric capacitors. This study provides a possible way to improve the reliability properties of ferroelectric devices in the non-volatile memory application.

**Electronic supplementary material:**

The online version of this article (10.1186/s11671-019-2970-6) contains supplementary material, which is available to authorized users.

## Background

Lithium niobate (LN) single-crystal films, due to their excellent physical properties, [1–6] have been widely used in surface acoustic wave oscillators, electro-optic modulators, and data storage based on the domain switching. Recently, wafer-scale lithium niobate-on-insulator (LNOI), which has great potential application for high-density integrated circuits in electro-optic, acousto-optic, and data storage devices, is fabricated by an ion implantation and wafer bonding technology. This technology allows for a wide variety of substrates, such as LN, silicon, and even the CMOS circuit [3, 7–9]. However, the imprint hysteresis loop originated from preferred orientations and the poor fatigue endurance of LN films, due to by-electrode charge injection, destabilize the retention of polarization reversal, which limits their application in non-volatile memory devices [10–13]. The preferred orientations related to interfacial passive layers formed between ferroelectric layers and electrodes, which can induce a strong depolarization field in the opposite direction of polarization. It can drive out the injected charges after the removal of the applied voltage or during intermittent time of the sequent pulse stressing [11, 12]. On the other hand, because of the presence of interfacial passive layers, the fatigue endurance of LN films will be improved by blocking the charge injection from by-electrode after ferroelectric switching. However, the fatigue process accelerates if the time of the applied pulse periodicity is shorted below 0.5 s. This is described by the interfacial passive layers contribution of the accumulative space charge at certain frequencies [11]. It is reported that an inlaid Al_2_O_3_ dielectric film can play as a tunnel switch in the dielectric/ferroelectric bilayer capacitor, for example, in Al_2_O_3_/Pb (Zr,Ti)O_3_, and Al_2_O_3_/Mn-doped BiFeO_3_ bilayer structures [14–16]. The Al_2_O_3_ tunnel switch turns on as a conductor during polarization switching, but switches off as an insulator to block the by-electrode charge injection after completed polarization switching or no switching operation [14]. Therefore, it can prevent the unwanted injected charges and polarization backswitching, and then improve the reliability of dielectric/ferroelectric bilayer capacitor.

In this paper, we fabricated 200-nm-thickness Z-cut 5% Mg-doped congruent LN single-crystal thin films and then deposited ultrathin Al_2_O_3_ layers with various thicknesses (2–6 nm) on LN to form bilayer capacitor structures. The Al_2_O_3_ films as tunnel switch layers can improve the fatigue endurance. Asymmetric electrodes (Au/Pt electrodes) are designed to form a built-in electric field against the depolarization field induced by the interfacial passive layers. The electrical results exhibit the symmetrisation of hysteresis loop transferred from the domain switching current transients with time. Meanwhile, it also proves that the inlaid Al_2_O_3_ layer plays as a tunnel switch layer, which can turn up during the ferroelectric switching and close after completed polarization switching or no switching operation.

## Methods

The Z-cut 5% Mg-doped congruent LiNbO_3_ (LN) single-crystal thin films were peeled off from their bulk crystals by using an ionic implantation and wafer bonding technology, as described elsewhere [10, 11, 17, 18]. In detail, the surface layer of a LN bulk crystal was first implanted with He ions in desired depth by controlling the implantation energy and the dose of injected ions, and then 5 nm Cr adhesion layer and 100 nm Pt bottom electrode layer are deposited by DC sputtering (K. J. Lesker PVD-75). The surface layer was bonded to another LN substrate covered with 1-μm-thick SiO_2_ buffer layer and sliced off. The thickness of LN film is controlled to about 200 nm by chemical mechanical polishing. Subsequently, ultrathin Al_2_O_3_ films with thicknesses (*d*) of 2–6 nm were deposited by ALD (TFS-200, Beneq, Finland). In detail, the precursor gases are diethyl zinc and de-ionized water. They were pulsed alternately into the reaction chamber with a pulse time of 50 ms and separated by purging steps using argon for 2 s at the reaction temperature of 200 °C [19]. Finally, top Au square electrodes with areas of 1.0 × 10^−4^ cm^2^ were deposited through a metal shallow mask.

The thicknesses of Al_2_O_3_ layers deposited on the Si wafer as contrast were measured by a spectroscopic ellipsometry system (GES-5E, SOPRA, Courbevoie, France). The film structure was analyzed by the X-ray diffraction (XRD) (Bruker D8 Advance) in a θ-2θ scanning mode with Cu *K*_α_ radiation as well as cross-sectional scanning electron microscopy (SEM, Sigma HD, Zeiss). To study the domain switching dynamics, several square pulses with a rising time of 10 ns were applied to top electrodes by using a single-channel Agilent 8114A pulse generator, where bottom electrodes were grounded. In the circuit, the domain switching current (*I*_sw_) across in-series internal resistors of all instruments with the total resistance was monitored using a LeCroy HDO6054 oscilloscope. The values of both output resistance of the pulse generator *R*_W_ and the input resistance of the oscilloscope *R*_O_ are 50 Ω, respectively.

## Results and Discussion

**Fig. 1:**
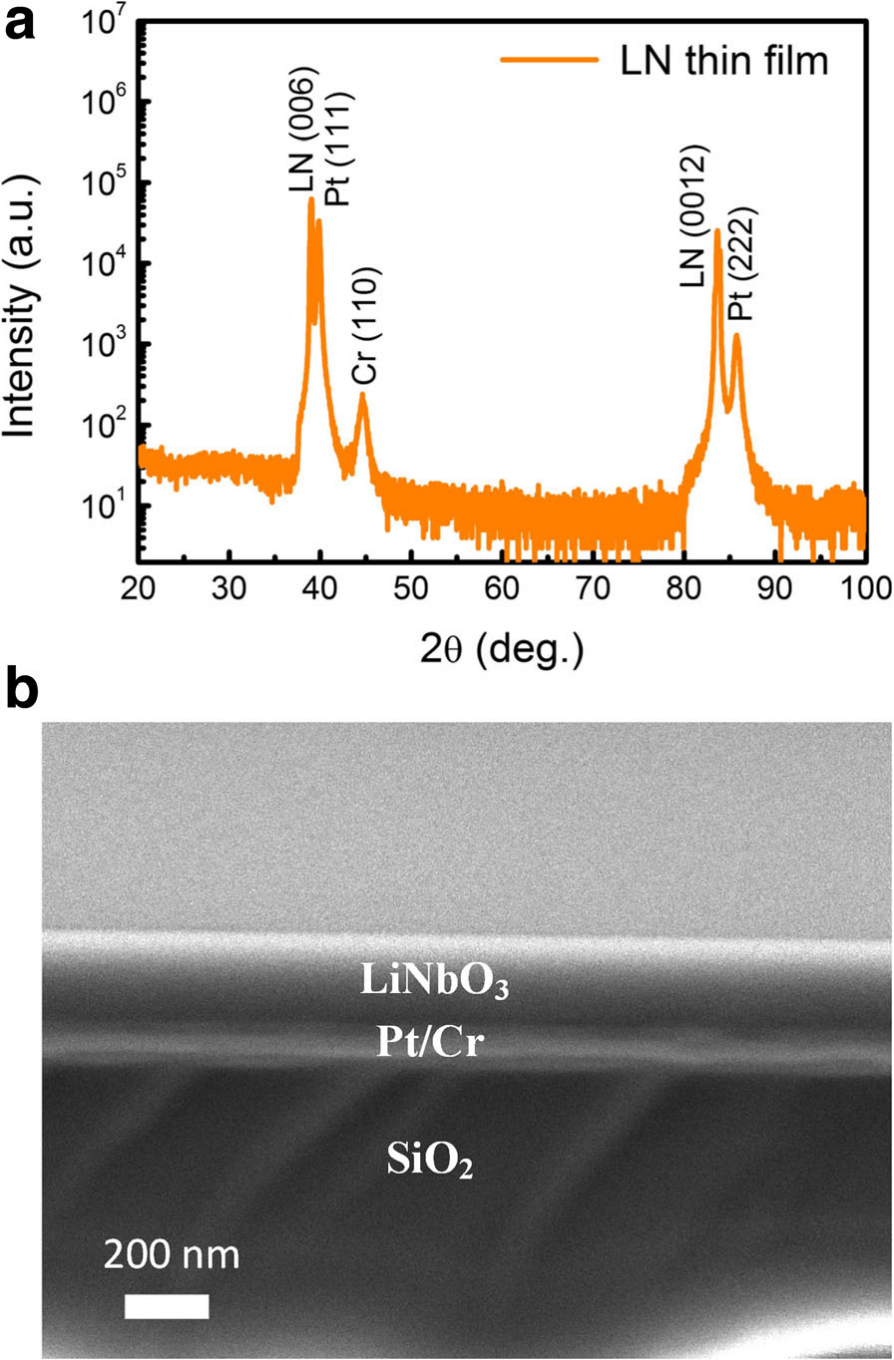
**a** The XRD pattern and **b** cross-section SEM image of the Z-cut 200-nm-thick LN/Pt/Cr/SiO_2_/LN film

Figure 1a shows the XRD result of the LN thin film on a Pt/Cr/SiO_2_/LN substrate. The film has strong (00 *l*) reflections indexed in the rhombohedral phase symmetry. In addition, there are also some diffraction peaks of Pt and Cr films marked in Fig. 1a. The absence of any other peaks confirms the high crystallinity of the LN film without phase impurity. The cross-sectional SEM image of the sample shown in Fig. 1b demonstrates the clear interface structure with LN, Pt, Cr, and SiO_2_ stacking layers.

**Fig. 2:**
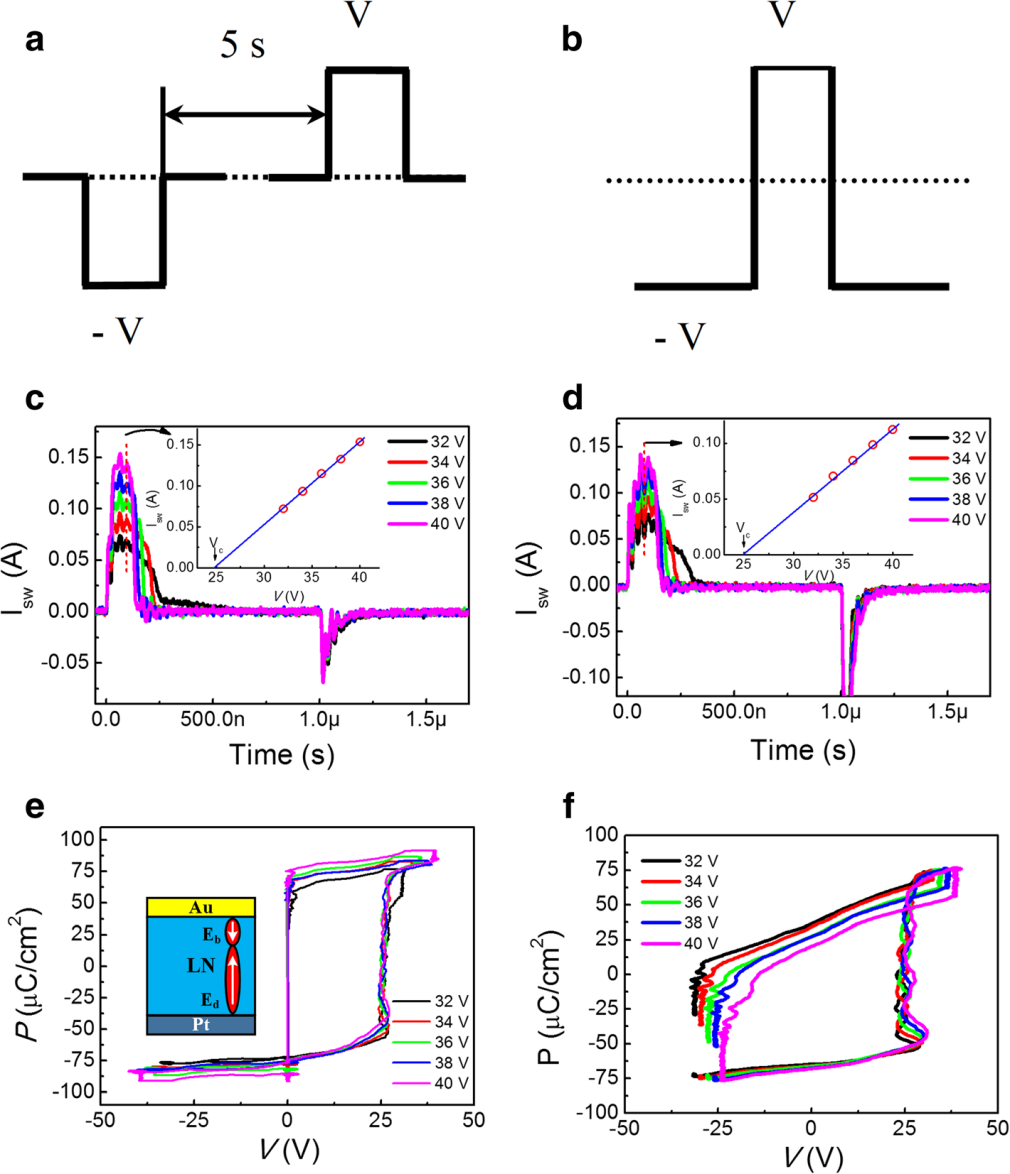
The sketch of the two sequence pulse voltage modes with **a** type I (double pulses in two opposite polarities) and **b** type II (a single switching pulse overlapping a negative DC bias). Domain switching current transients under different *V* applied to a virgin sample in **c** type I and **d** type II modes, where the insets show the linear fit of the dependence of the plateaus of domain switching current on *V*. *P*-*V* hysteresis loops under different *V* transferred from domain switching current transients in **e** type I and **f** type II. Schematic diagram of the Au/LN/Pt structure and the directions of the built-in electric field *E*_b_ and depolarization field *E*_d_ in the inset of **e**

In order to study the domain switching kinetic mechanism, two types of pulse voltage modes are designed as clearly shown in Fig. 2a and b [11]. Type I is configured as double pulses in opposite polarities with the time interval of 5 s. The first pulse is applied to switch the upward polarization state pointing to the top electrode and the second one can switch the downward polarization. However, limited by programming time of a single-channel pulse generation, the minimum time interval is too long to catch the domain switching current transient invoked by the second pulse, due to the preferred domain orientation. To catch the domain switching current transient, a single pulse overlapping a negative baseline DC bias is proposed in type II, where the initial negative DC bias can switch the upward polarization state and the positive pulse sets the domain downward. Here, the width of the two type pulses is set to1 μs.

Figure 2c and d show the domain switching current transients versus time (*t*) of Au/LN/Pt structure sample under various applied voltages (*V*) in type I and type II modes, respectively. The plateaus of domain switching current transients are observed that narrow in width but increase in height with *V* increasing after the initial capacitor charging current at 30 ns. The height of plateau in two modes both shows a linear relationship with the increase of *V* and the results are summarized in the insets by the solid-line fitting of the data [11, 13]. The coercive voltage (*V*_c_) value in the two modes can be derived to about 24.7 V from the line interception with the voltage axis. After the termination of the switching pulse, the capacitor discharging current occurs after 1 μs, which suggests that the preferred domain orientation is the upward polarization state pointing to the top electrode.

*P*-*V* hysteresis loops under different applied voltages in two type modes can be transferred directly from the corresponding domain switching current transients in Fig. 2c and d, and the results are shown in Fig. 2e and f, respectively [11, 20]. A determined forward coercive voltage of about 25 V invariable with *V* is obtained in the two types pulses. The coercive voltage approaches to *V*_c_ extracted from the linear *I*_sw_-*V* plot in the inset of Fig. 2c and d. Unlike the non-doped LN film, the *V*_c_ is variable and the value is equal to the maximum applied voltages [10]. For the 5% Mg-doped LN, the defined *V*_c_ is invariable with *V*, as shown in Fig. 2e and f. This is because the Mg doping can generate Li-site metal vacancies and oxygen vacancy-related defects, [21–23] which can trap space charges and effectively shorten the resistance degradation time across the interfacial layers between the film and top/bottom electrodes [11]. Therefore, domain switching currents overlap with capacitor charging currents in acceleration of domain switching speed with a definite *V*_c_, as shown in Fig. 2c and d. However, limited by the pulse generator, the output baseline voltage in type II mode cannot be shift symmetrically when increasing applied pulse voltage over 32 V. Compared to the imprinted loops along the positive voltage axis in Fig. 2e, the symmetrisation of the loops are achieved along the voltage axis in Fig. 2f, different from those in Pt/LiNbO_3_/Pt structures where the *P*-*V* hysteresis loops in either type I or type II are imprinted toward a positive voltage [11]. The reason of the symmetrical *P*-*V* loops in Fig. 2f may be attributed to the designed asymmetric electrodes (here Au/Pt). The work function of Au electrode is 5.1 eV, which is slightly smaller than that of Pt (5.65 eV) [24]. There will induce a built-in electric field (*E*_b_) with the direction pointing from the top electrode to the bottom electrode, shown in the inset of Fig. 2e. The depolarization field (*E*_d_) induced by the interfacial passive layers has the opposite direction to *E*_b_. The *E*_d_ can switch back the polarization in a very short time after the termination of the switching pulse in type II for the symmetrical electrodes (Pt/Pt) [11]. In our experiment, the *E*_b_ can partially screen the *E*_d_ and accumulate injected charges in compensation of an internal imprint field, [16] which can slow down the backswitching time. Hence, the switched domain can maintain and backswitching current transient will be captured by type II pulse. However, the time interval of the two pulses with opposite polarities in type I mode is too long. After the first pulse, the trapped injected charges by *E*_b_ will be gradually driven out of the film by *E*_d_ before the arrival of the second pulse in type I [11]. In order to prove the attribution of built-in electric field to the symmetrisation of the loops, Pt/LiNbO_3_/Pt symmetrical structure sample was prepared and the imprinted loops along the positive voltage axis were transferred directly from the corresponding domain switching current transients in Additional file 1: Figure S1a at positive pulse with voltages/widths of 30–40 V/500 ns, shown in Additional file 1: Figure S1b.

**Fig. 3:**
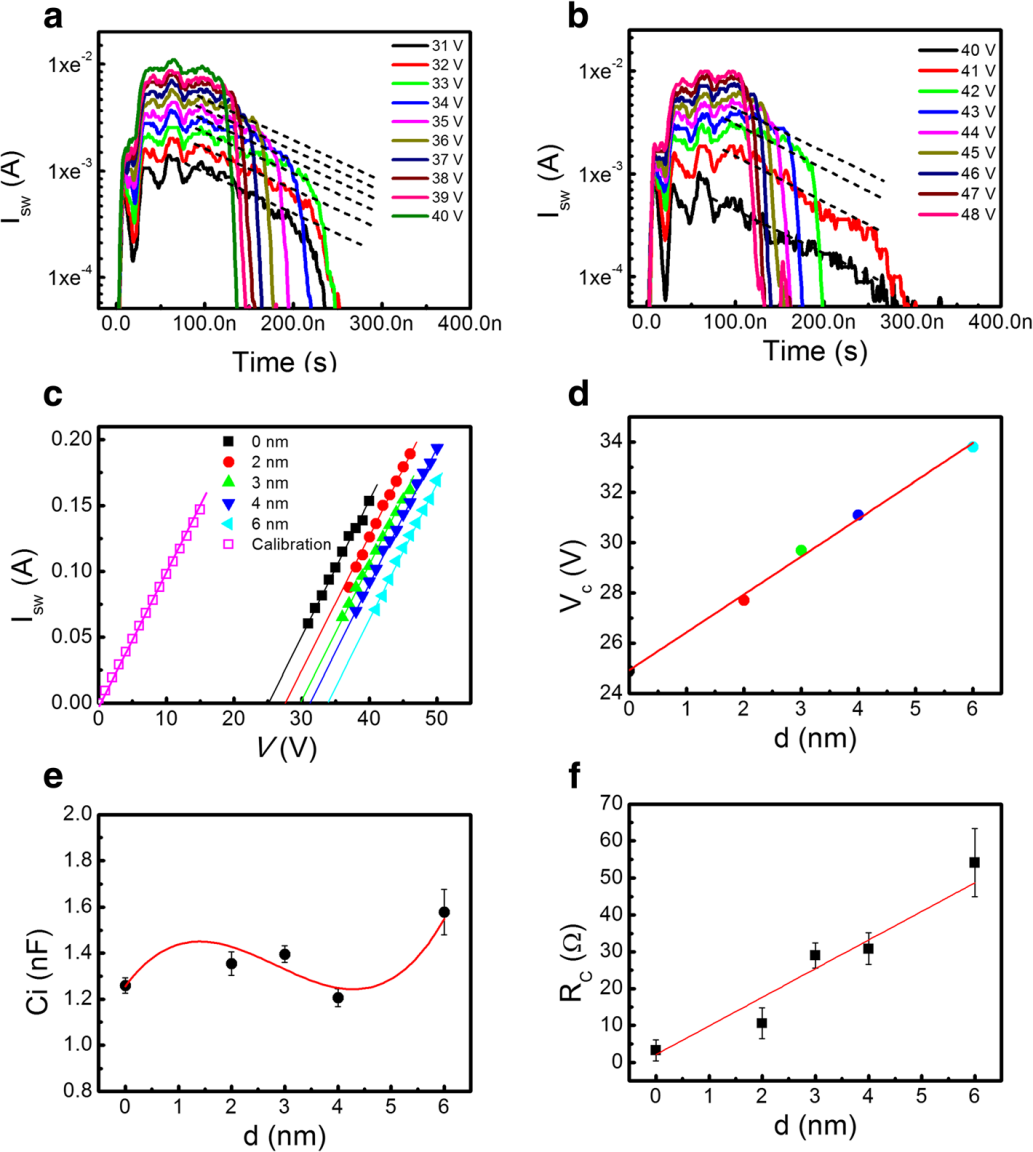
**a**, **b** *I*_sw_-*t* dependences in type I under different *V* applied to the Al_2_O_3_/LN bilayer with the Al_2_O_3_ thickness *d* = 0 and 6 nm, respectively, fitted by a series of parallel dotted lines to Eq. (1). **c** The plateaus of domain switching current as a function of the applied voltage with different Al_2_O_3_ layer thicknesses, where the solid lines show the best fit of the data to Eq. (2). **d** The Al_2_O_3_-layer-thickness *d* dependence of the coercive voltage (*V*_c_) extracted from **c**. **e**, **f** The extracted interfacial capacitance *C*_i_ and contact resistance *R*_C_ as functions of the Al_2_O_3_ layer thickness *d*

Figure 3a and b show the domain switching current (*I*_sw_) transients versus time (*t*) of LN and Al_2_O_3_ (6 nm)/LN samples under different applied voltage (*V*) in type I mode. After the plateau of domain switching, the switching current *I*_sw_ decays and is given by: [13]

1 $$ {I}_{\mathrm{sw}}={I_{\mathrm{sw}}}^0\exp \left(-\frac{t-{t}_0}{R_{\mathrm{L}}{C}_{\mathrm{i}}}\right)\ \left({t}_0\le t\le {t}_{\mathrm{sw}}\right) $$

where *t*_0_, *t*_sw_, *R*_L_, and *C*_i_ are the beginning time of domain switching, the completion time of domain switching, the total resistance of all the in-series resistors in the circuit, and the interfacial non-ferroelectric capacitance, respectively. This describes the charge trapping effect which can be modeled as an interfacial passive layer in series with an ideal ferroelectric layer. *I*_sw_^0^ is defined as switching current and is given by:

2 $$ {I_{\mathrm{sw}}}^0=\frac{V-{V}_{\mathrm{fc}}}{R_{\mathrm{L}}} $$

During domain switching, the voltage applied on the ferroelectric layer is fixed at the coercive voltage *V*_fc_, and the extra voltage (*V*-*V*_fc_) is applied to *R*_L_. *R*_L_ also included the circuit parasitic resistance (*R*_P_) and contact resistance (*R*_C_) between the film and electrodes; hence, *R*_L_ = *R*_O_ + *R*_W_ + *R*_P_ + *R*_C_. The decayed part of the switching current transients versus time can be fitted by Eq. (1). The time constant *R*_L_*C*_i_ can be estimated from the slope of the fitted lines. Figure 3c shows *I*_sw_^0^-*V* plots with different Al_2_O_3_ thicknesses. *R*_L_ and *V*_C_ were estimated from the slopes and the *X*-axis intercept of the linear fitted lines. It can be seen that the *V*_C_ is increased linearly with increasing the Al_2_O_3_ thickness *d*, as shown in Fig. 3d. Here, the *C*_i_ values were estimated as the error bounds at each *V* in Fig. 3e [13]. The results show that *C*_i_ value almost kept constant (1.4 ± 0.2) nF with increasing Al_2_O_3_ layer thickness from 0 to 6 nm.

In order to calculate *R*_C_, the top and bottom electrodes are shorted, which can obtain the *R*_P_ (~ 2 Ω) with different applied voltages, shown as the circuit calibration by the opened symbols in Fig. 3c. Therefore, the *R*_C_ corresponding to *d* is calculated and the result is showed in Fig. 3f. *R*_C_ increases linearly from 3 ± 2.5 Ω at *d* = 0 to 55 ± 10 Ω at *d* = 6 nm. The almost *d*-independent large *C*_i_ values suggest that the Al_2_O_3_ layer works as a series resistor during domain switching. This means that the Al_2_O_3_ tunnel switch was switched on during FE switching.

**Fig. 4:**
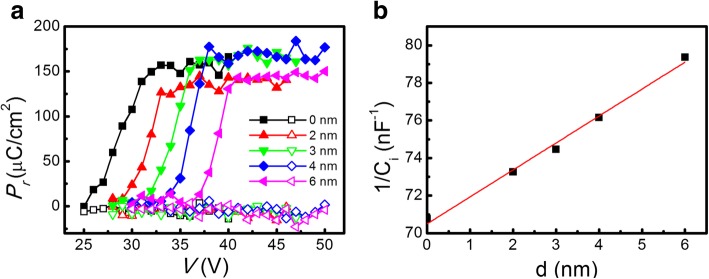
**a** The switching (*P*_sw_) and nonswitching (*P*_nsw_) polarizations versus *V* with *d* increased from 0 to 6 nm under pulses in type I mode. **b** The Al_2_O_3_-layer-thickness *d* dependence of 1/*C*_tot_ measured by an impedance analyzer at 100 kHz

In order to obtain the total capacitance of the bilayer during FE nonswitching, the switching (*P*_sw_) and nonswitching (*P*_nsw_) polarizations vesus *V* with *d* increased from 0 to 6 nm under pulses in type I mode are measured and the result are showed in Fig. 4a. The purpose of choosing the type I pulse is to obtain the curve of *P*_nsw_-*V* when the direction of the applied voltage is consistent with the polarization orientation, from which the total capacitance (*C*_tot_) of the bilayer can be calculated from the relation, *C*_tot_ = *S*·*dP*_nsw_/*dV*, where *S* is the electrode area. It can totally exclude the charge effects by FE switching in the type I pulse mode, but using the type II mode cannot achieve this effect with the negative switching polarization, which can switch back the polarization involved with the charges injection. The difference between *P*_sw_ and *P*_nsw_ is 2Pr, as shown in Fig. 4a. It has small change with *d* from 0 to 6 nm, whereas the *P*_nsw_ (open symbols) signals are too weak to be monitored by an oscilloscope. To prove the Al_2_O_3_ tunnel switch layer working as a dielectric capacitor, the direct *C*_tot_ measurements using a low-frequency impedance analyzer at 100 kHz with no additional DC bias were carried out and their results are shown in Fig. 4b, which can be fitted by Eq. (3):

3 $$ \frac{1}{C_{\mathrm{tot}}}=\frac{1}{C_{\mathrm{f}}}+\frac{d}{\varepsilon_0{\varepsilon}_{\mathrm{Al}}S} $$

where *ε*_Al_ is the dielectric constant of the Al_2_O_3_ layer and *ε*_0_ is the vacuum permittivity of free space. *C*_f_ and *S* represent the capacitance of the ferroelectric layer and the electrode area, respectively. Figure 4b shows the linear 1/*C*_tot_ versus *d* plot, which suggests that the Al_2_O_3_ layer becomes a highly insulating dielectric film under nonswitching situation or post-switching. It can be derived that *C*_f_ ≈ 14 pF and *ε*_Al_ ≈ 7.9 from Eq. (3). Therefore, the interposed thin Al_2_O_3_ layer is proved as a dielectric capacitor. During FE nonswitching as well as after FE switching, the Al_2_O_3_ tunnel switch closes as an insulator.

**Fig. 5:**
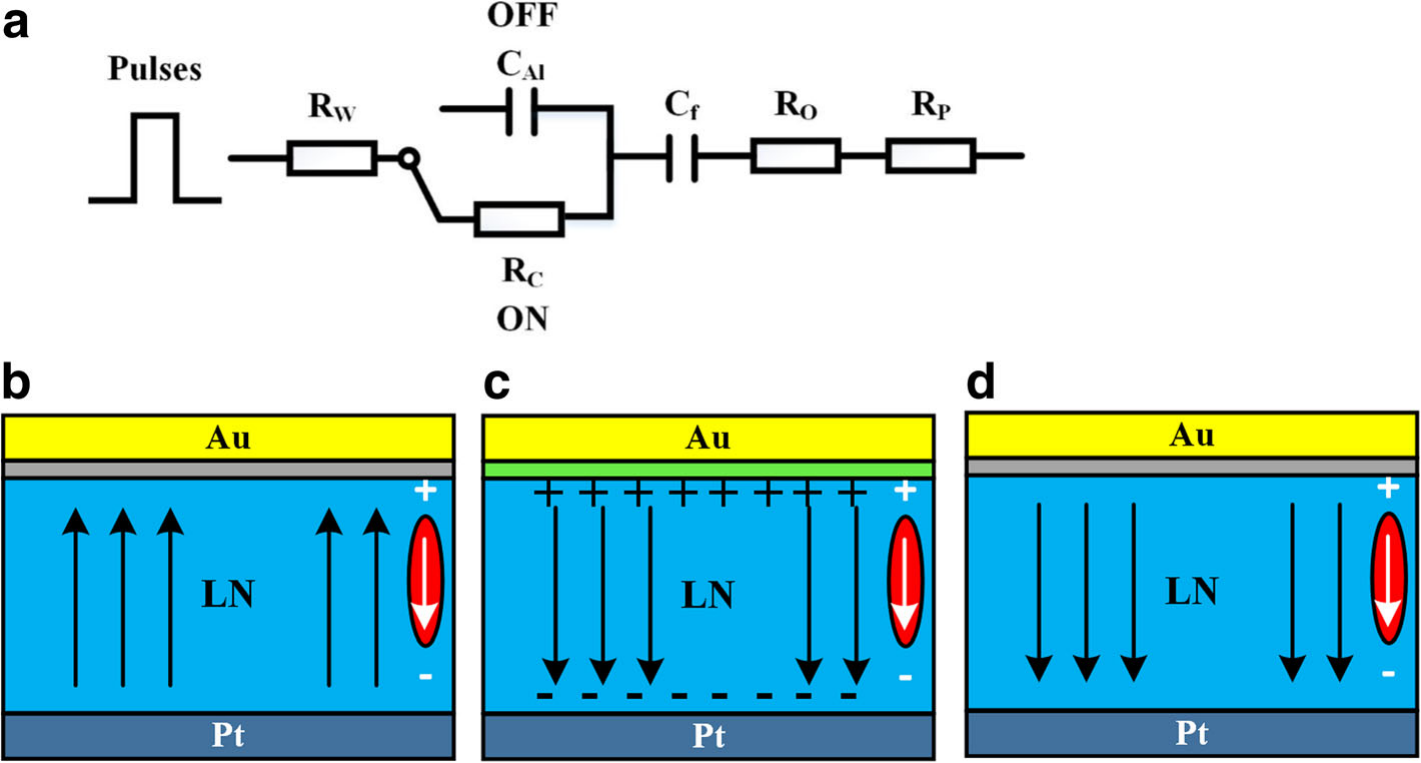
Schematic diagrams of the Al_2_O_3_/LN bilayer structure switched in type I or type II. **a** The sketch of the equivalent on-off circuit of the in-series resistors and capacitors for the Al_2_O_3_ tunnel switch. **b** Initial preferred polarization orientation and built-in electric field; **c** The Al_2_O_3_ tunnel switch turning on and domain switching; **d** The Al_2_O_3_ tunnel switch switching off and polarization maintain

Figures 5 show schematic diagrams of the Al_2_O_3_/LN bilayer structure switched in type I or type II mode. Figure 5a sketches the equivalent on-off circuit of the in-series resistors and capacitors for the Al_2_O_3_ tunnel switch. In the initial state, as shown in Fig. 5b, the preferred polarization orientation is the upward polarization state pointing to the top electrode. The built-in electric field induced by the asymmetric electrodes directs from Au electrode to Pt electrode. When applying the polarization voltage, the FE switching occurs. It is understood that the voltage is applied inversely proportional to the capacitance in the circuit. In Al_2_O_3_/LN bilayer structure, during the FE switching, the LN layer has a large capacitance. Therefore, most of the external applied voltage applies on the Al_2_O_3_ layer. Ultrathin Al_2_O_3_ layer is injected by electrode charge. It switches on as a resistor when the applied voltage exceeds the Al_2_O_3_ tunneling threshold, as shown in Fig. 5c. After the completion of FE switching or for the case of a nonswitching situation, the capacitance of LN layer is very small and the applied voltage on Al_2_O_3_ decreases lower than the tunneling threshold voltage. At this moment, the Al_2_O_3_ layer plays as an insulator and switches off, as shown in Fig. 5d.

**Fig. 6:**
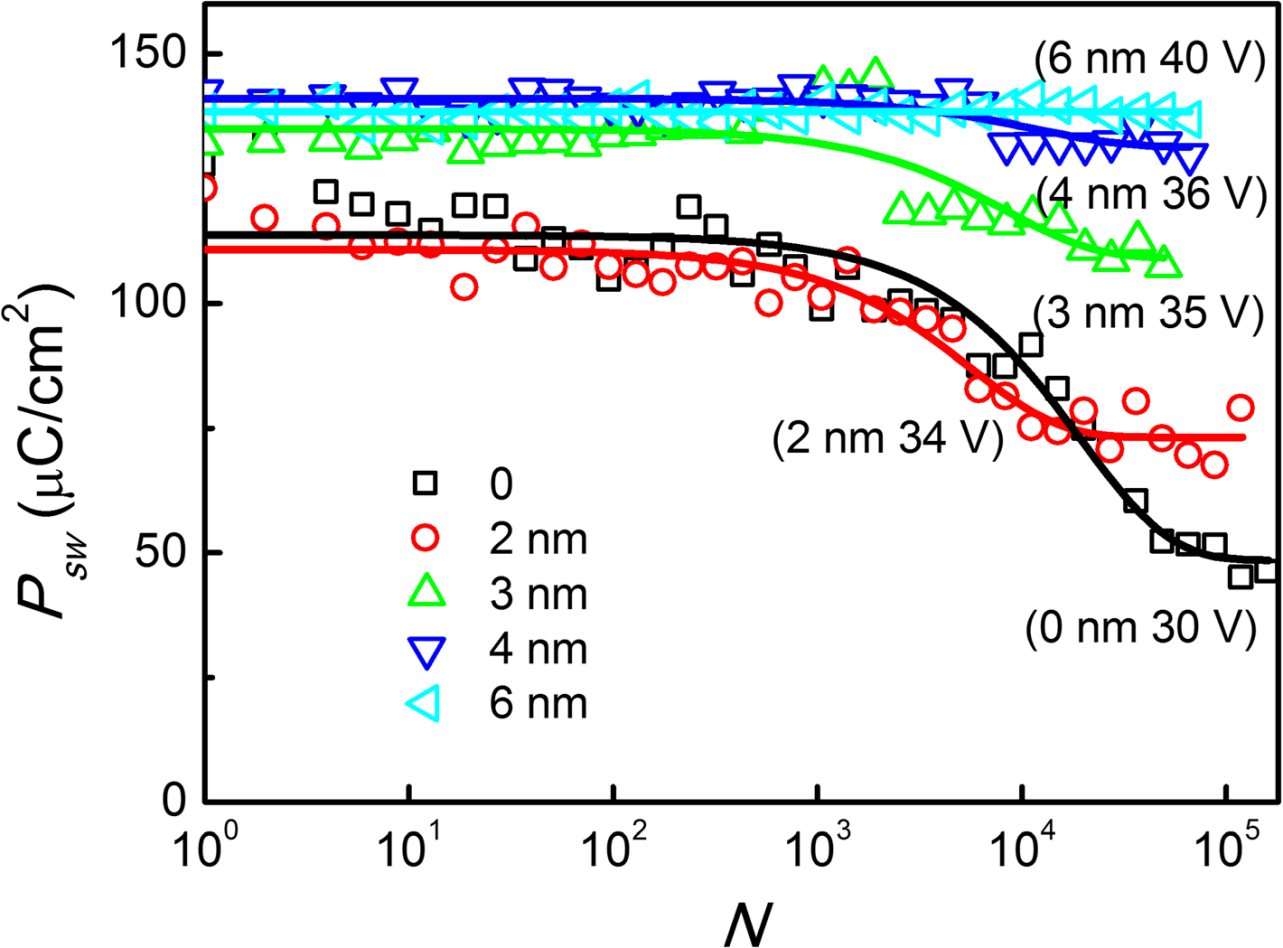
Cycling number dependences of switched polarizations in Al_2_O_3_/LN bilayer structure with the thickness of Al_2_O_3_ ranging from 0 to 6 nm under over 10^4^ cycles of pulse stressing. The width of pulses is 1000 ns in the periodicity of 0.5 s

Figure 6 shows the cycling number dependences of switched polarizations in Al_2_O_3_/LN bilayer structure with the thickness of Al_2_O_3_ ranging from 0 to 6 nm in type I mode. The width of pulses is 1000 ns with a periodicity of 0.5 s. It can be clearly seen that the fatigue endurance of the Al_2_O_3_/LN bilayer structure is improved gradually with increasing the Al_2_O_3_ thickness with over 10^4^ cycles of pulse stressing. The fatigue property in type II mode is similar to the result in type I mode, which was showed in Additional file 1: Figure S2 of supporting information. Unfortunately, the electrical breakdown would occur easily in the type II mode after longtime DC voltage applied with near 10^4^ cycles of pulse stressing. The data can be fitted using the model for the coexistence of domain-wall pinning and depinning within each cycle, as shown by the solid lines in Fig. 6, where the fatigue physics was attributed to by-electrode charge injection [13]. When the Al_2_O_3_ layer inserted between the Au electrode and LN layer, it can block the by-electrode injection charge path and improve the fatigue endurance. However, in the bilayer structure, some issues should be further considered. For example, with increasing the thicknesses of Al_2_O_3_ from 0 to 6 nm, the coercive voltage enlarged from near 25 to 34 V, which can be reduced by improving the quality of the Al_2_O_3_ layer. Actually, a few atomic layers of Al_2_O_3_ with high quality or less defect can effectively block the charges injected by electrodes, which is confirmed elsewhere by optimizing atomic layer deposition processing conditions (such as temperature and time) [25].

Recently, ferroelectric domain-wall memories based on the erasable conducting charged domain walls and the non-destructive electrical read-out of the polarization states have been proposed in our following research work [26, 27]. Large conductivity of charged domain walls in lithium niobate single crystals is obtained after domain switching [28, 29]. Therefore, the thinner lithium niobate single crystal thin films on silicon substrates are the promising materials for integrated ferroelectric domain-wall memories and its retention and fatigue endurance properties can be improved by design of Al_2_O_3_/lithium niobate bilayer.

## Conclusions

Two hundred nanometer LiNbO_3_ single-crystal films with 5% Mg-doping were prepared by ion slicing of surface layers from bulk LN single crystals, and then the ultrathin Al_2_O_3_ films with thicknesses ranging from 2 to 6 nm as tunnel switch layers were deposited on 5% Mg-doped LN film to form bilayer structures by atomic layer deposition. The symmetrized *P-V* hysteresis loops along the voltage axis are observed under applied pulse voltages in type II mode, which may be attributed to the built-in electric field induced by asymmetric electrodes in Au/LiNbO_3_/Pt and compensation of the internal imprint field. The domain switching current (*I*_sw_) transients and its transferred *P-V* hysteresis loops reveal that the ultrathin Al_2_O_3_ layer plays as an idea tunnel switch. It turns on during FE switching, but closes during the nonswitching or after FE switching, minimizing the adverse interference with FE switching. Furthermore, the fatigue endurance of the FE capacitor is improved gradually with increasing the tunnel switch layer thicknesses from 2 to 6 nm. The Al_2_O_3_/LN bilayer structure paves the way to design robust ferroelectric devices in alleviating the fatigue problem by-electrode charge injection.

## Additional file

Additional file 1: The imprinted P-V hysteresis loops of Pt/LiNbO_3_/Pt symmetrical structure sample in type II mode and the fatigue property of Al_2_O_3_/LiNbO_3_ bilayer structure in type II mode. (DOCX 144 kb)

## Abbreviations

ALD: Atomic layer deposition
CMOS: Complementary metal oxide semiconductor
FE: Ferroelectric
LN: Lithium niobate
SEM: Scanning electron microscopy
XRD: X-ray diffraction
