# Supplementary material for: Improved Ferroelectric Performance of Mg-Doped LiNbO3 Films by an Ideal Atomic Layer Deposited Al2O3 Tunnel Switch Layer
Source: Nanoscale Res Lett. 2019 Apr 16;14:131. doi: 10.1186/s11671-019-2970-6 (PMC6468035; doi:10.1186/s11671-019-2970-6)
Supplement: Supplementary file 1 — The imprinted P-V hysteresis loops of Pt/LiNbO3/Pt symmetrical structure sample in type II mode and the fatigue property of Al2O3/LiNbO3 bilayer structure in type II mode. (DOCX 144 kb) [file 11671_2019_2970_MOESM1_ESM.docx]

**Supporting Information (**NARL-D-19-00088**)**

**Improved ferroelectric performance of Mg-doped LiNbO_3_ films by an ideal atomic layer deposited Al_2_O_3_ tunnel switch layer**

Yan Zhang^1^, Qing Hua Ren^2*^, Xiao Jie Chai^1^, Jun Jiang^1^, Jian Guo Yang^1^, An Quan Jiang^1*^

^1^ State Key Laboratory of ASIC & System, School of Microelectronics, Fudan University, Shanghai 200433, China.

^2^ State Key Laboratory of Functional Materials for Informatics, Shanghai Institute of Microsystem and Information Technology, Chinese Academy of Sciences, Shanghai 200050, China.

[15110720078@fudan.edu.cn](mailto:15110720078@fudan.edu.cn), [*qhren@mail.sim.ac.cn](mailto:*qhren@mail.sim.ac.cn), [17112020013@fudan.edu.cn](mailto:17112020013@fudan.edu.cn), [12110720035@fudan.edu.cn](mailto:12110720035@fudan.edu.cn), [13110720062@fudan.edu.cn](mailto:13110720062@fudan.edu.cn), [*aqjiang@fudan.edu.cn](mailto:*aqjiang@fudan.edu.cn)

**Contents**

1, Domain switching current transients and imprinted *P*-*V* hysteresis loops of Pt/LiNbO_3_/Pt symmetrical structure sample in type II mode with voltages/widths of 30-40 V/500 ns





**Figure S1** **a** Domain switching current transients under different *V* applied to Pt/LiNbO_3_/Pt symmetrical structure sample in type II mode with voltages/widths of 30-40 V/500 ns; **b** *P*-*V* hysteresis loops under different *V* transferred from domain switching current transients **a**.

2, Cycling number dependences of switched polarizations in Al_2_O_3_/LN bilayer structure with the thickness of Al_2_O_3_ ranging from 0 to 6 nm in type II mode.





**Figure S2** Cycling number dependences of switched polarizations in Al_2_O_3_/LN bilayer structure with the thickness of Al_2_O_3_ ranging from 0 to 6 nm in type II mode under over 10^4^ cycles pulses stressing. The width of pulses is 1000 ns in periodicity of 0.5 s.
